# Indirect adaptive soft computing based wavelet-embedded control paradigms for WT/PV/SOFC in a grid/charging station connected hybrid power system

**DOI:** 10.1371/journal.pone.0183750

**Published:** 2017-09-06

**Authors:** Sidra Mumtaz, Laiq Khan, Saghir Ahmed, Rabiah Bader

**Affiliations:** Department of Electrical Engineering, COMSATS Institute of Information Technology, Abbottabad, KPK, Pakistan; Chongqing University, CHINA

## Abstract

This paper focuses on the indirect adaptive tracking control of renewable energy sources in a grid-connected hybrid power system. The renewable energy systems have low efficiency and intermittent nature due to unpredictable meteorological conditions. The domestic load and the conventional charging stations behave in an uncertain manner. To operate the renewable energy sources efficiently for harvesting maximum power, instantaneous nonlinear dynamics should be captured online. A Chebyshev-wavelet embedded NeuroFuzzy indirect adaptive MPPT (maximum power point tracking) control paradigm is proposed for variable speed wind turbine-permanent synchronous generator (VSWT-PMSG). A Hermite-wavelet incorporated NeuroFuzzy indirect adaptive MPPT control strategy for photovoltaic (PV) system to extract maximum power and indirect adaptive tracking control scheme for Solid Oxide Fuel Cell (SOFC) is developed. A comprehensive simulation test-bed for a grid-connected hybrid power system is developed in Matlab/Simulink. The robustness of the suggested indirect adaptive control paradigms are evaluated through simulation results in a grid-connected hybrid power system test-bed by comparison with conventional and intelligent control techniques. The simulation results validate the effectiveness of the proposed control paradigms.

## 1 Introduction

The global warming and environmental deterioration are considered as a factor of supply and consumption of energy based on the fossil fuels. The fastest growing electrical energy demand, continuously diminishing fossil fuel energy sources and pollution crises bring renewed interest in renewable energy. Renewable energy is clean, sustainable and inexhaustible.

Among numerous renewable energy sources, wind energy conversion system (WECS) and PV energy conversion system (PVECS) are the fastest growing energy sources. In WECS, the wind turbine blades are used to harvest the kinetic energy of wind. The kinetic energy is converted to electrical power by using an appropriate generator. WECS can be categorized into fixed-speed generation and variable-speed generation. The variable-speed WECS has several advantages as compared to fixed-speed WECS in-terms of operation, efficiency and power quality [1]. The use of a direct-drive permanent magnet synchronous generator (PMSG) with a wind turbine, significantly enhances the reliability of the VSWT-PMSG, because, the absence of magnetizing current in PMSG allows it to operate at high power factor [2]. The PVECS converts the solar energy into electrical energy. The power generation capability of WECS and/or PVECS is heavily dependent on local weather patterns, i.e., wind speed, irradiance and temperature. To improve the output efficiency of WECS and PVECS, it is crucial to operate WECS and PVECS near maximum power point (MPP).

For WECS, Optimal torque control (OTC) MPPT strategy is quite popular, as it extracts the optimum wind energy [3], [4]. In OTC MPPT method, the torque of PMSG at the given wind speed is amended on the basis of the maximum power reference torque. Although, the OTC MPPT strategy is simple and fast but the efficiency of this method is low, because, the wind speed is not measured directly. Therefore, the wind fluctuations cannot be captured substantially and abruptly on the reference signal [5]. Tip speed ratio (TSR) is another commonly used MPPT technique for WECS. The optimal TSR remains constant irrespective of wind speed and guarantees that the maximum power is extracted [6]. Although, TSR MPPT scheme is simple as it continuously and directly measures the wind speed but in reality, measuring the accurate wind speed becomes challenging and also, increases the cost of the system. In power signal feedback (PSF) MPPT for WECS, the wind turbine reference optimum power curve is obtained through experimental results. Then, the data of MPPs versus wind speeds are used from a lookup table [7]. However, the PSF MPPT scheme is complex to implement. In perturbation and observation (P&O) or hill climbing (HC) MPPT for WECS, the control variable is perturbed with a small step-size and the subsequent variations are observed in the objective function till the slope becomes zero [8]. The main disadvantage of P&O or HC MPPT scheme is that the indistinct difference between the powers results in incorrect decision in defining the direction for next step. The intelligent MPPT controls for WECS are also reported in the literature. The intelligent MPPT controls include artificial neural network (ANN) [9], fuzzy logic [10] and NeuroFuzzy [11]. Though, the neural network has the strong capability of learning but it needs a tremendous amount of training data and requires a long time to train the network. The fuzzy logic controllers have high convergence speed and acceptance of ill-defined signals but their design depends upon trial-and-error method. The NeuroFuzzy MPPT is successfully implemented to acquire the maximum power from WECS. However, the inherent shortcoming of the NeuroFuzzy system is that it has long computational time and becomes trapped in local minima of the search space [12].

There are two main categorizes of MPPT control schemes for PV named as conventional and soft computing. Conventional MPPT control schemes include P&O, HC and incremental conductance (IC), whereas, the soft computing MPPT include ANN, fuzzy logic and evolutionary algorithms. The efficiency of the P&O MPPT is improved by subsiding the steady-state oscillation and reducing the probability of losing its tracking direction [13]. However, a boundary condition is applied to confine the MPP voltage in the range of the MPP locus. The HC MPPT control scheme is successfully applied for stand-alone parallel PV power generation [14]. However, the dynamic performance of HC suffers the divergence of MPP. The IC MPPT control scheme has a fast and accurate response but the perturbation step and marginal error are computed on the basis of trial and error approach [15]. The ANN based MPPT method is implemented to spontaneously identify the global MPP based on the preselected number of power measurements [16]. Although, the results yield the robustness of the proposed MPPT control scheme but at the cost of long computational time. In [17], two types of fuzzy logic controllers are implemented to track the PV MPP. One is an adaptive fuzzy logic controller and other is the conventional fuzzy logic controller. However, the adaptive fuzzy logic controller still requires fine tuning via trial and error, because, it exhibits the same behavior as that of the conventional fuzzy logic controller. A wavelet-based NeuroFuzzy control scheme is used to track the MPP of PV [18], [19]. Wavelets are introduced in the structure of NeuroFuzzy to improve the performance. The simulation results reveal high efficiency and fast response of wavelet-based NeuroFuzzy control scheme. The most popular evolutionary algorithms used to track the MPP of PV are the genetic algorithm (GA) [20], particle swarm optimization (PSO) [21] and differential evolution (DE) [22]. Although, evolutionary algorithms are the stochastic methods which are quite efficient to solve a real valued, nonlinear, multi-modal optimization problems. But the appropriate selection of control parameters, initial values, solution archive and locality of the search space are still the potential areas of concern.

SOFC is versatile, efficient and alternative energy source which generates electrical power directly from hydrocarbon fuels at 800–1000°C. The limitation of SOFC is that it has dynamic load following issue. In the case of large power variations, the hydrogen starvation occurs owing to the slow fuel supply which results in voltage drop, anode oxidation, and catalyst corrosion. In literature, the load following issue of SOFC is addressed by using two different control strategies. In one control strategy, the input hydrogen which is directly proportional to the SOFC stack current is controlled to resolve the load following issue [23]. In the other control strategy, the SOFC terminal voltage is maintained at a constant value to get the swift response of SOFC [24]. To address the load following issue of SOFC, different conventional and advanced control strategies are available. A feedforward controller is designed to resolve the fuel starvation problem but the system is not very efficient due to the weaknesses of feedforward controller, i.e., sensitive to external disturbances and the occurrence of steady state error [25]. In [26], a multi-loop feedforward/feedback control scheme is presented. Although, the plant is well stable under a tight linear region of operation but the overall system becomes complex and complicated. A master control PID feedback approach is adopted for load following but large load variations need more effective control scheme [27]. Model predictive control (MPC) is a predictive model and receding horizon optimization based feedback control system. This is an attractive approach for SOFC, because, a wide-range constraints of input/output variables of a nonlinear system are directly handled [28]. However, MPC is complex and computationally slow to get a reliable and accurate first-principles prediction model for a large-scale nonlinear system. Likewise, the advanced control methods are also used for the SOFC system which includes ANN predictive control [29] and fuzzy logic predictive control [30]. The effectiveness and stability of ANN predictive control and fuzzy logic predictive control is satisfactory. However, the resulting controllers are too convoluted and increase the cost of the system for hardware implementation.

The hybrid power system (HPS) is an emerging power generation scheme, because, the integration of renewable energy sources along with the storage systems improve the efficiency and energy supply, and its environmental and economic sustainability. A PV and SOFC along with electrolyzer based HPS is developed for supplying the electricity to residential load [31]. However, the stated HPS has small scale application. The dynamic operation and control of PV, wind and SOFC based HPS is presented [32]. A proficient power sharing method for all energy sources is also presented. However, the system is unable to store the surplus power produced by renewable energy sources. The dynamic performance of a stand-alone wind-solar-battery based HPS is investigated [33]. However, the HPS is not connected to the utility grid. A stand-alone PV, fuel cell and ultra-capacitor based HPS for the residential load is presented in [34]. The sizing and designing of PV, fuel cell and ultra-capacitor are also addressed to enhance the performance of the HPS. But the application of stated HPS is stand-alone. The dynamic modeling and control of a grid-connected wind–PV–battery based HPS is designed with versatile power transfer [35].The HPS is stable and has the dispatch-ability to provide the power to the grid. However, a small scale application of HPS is presented. The solar, wind and diesel engine based stand-alone HPS is used to meet the load [36]. However, the quality of power is not addressed. A novel operation and control strategy for a stand-alone HPS with battery storage is presented [37]. Different wind and load conditions are investigated. The optimum power from wind is extracted using machine side converter. A boost converter is used to control fuel cell and a buck converter is used to control electrolyzer. The stated HPS prevents the blackouts. However, the stand alone application of HPS is offered. A PV-FC-WES based grid-integrated HPS is used to meet the load [38]. The P&O algorithm is used to extract the MPP from PV. A pitch angle controller is used to extract the MPP from wind turbine. However, the load power is still unmanageable, therefore, local power shedding is used. The stochastic energy management is applied for a PV power based smart home with plug-in electric vehicle (PEV) to minimize the consumer’s energy charges [39]. By using the stochastic dynamic programming (SDP), home demand as well as the PEV charging requirements are satisfied. The modeling of PEV energy storage is reported for the first time. However, only a single home is considered. A PV array, battery storage system and the utility grid are used to provide the power to a building’s electrical loads [40]. The PV and battery are coupled to a DC bus. A DC/AC inverter connects the DC bus to AC loads and with the utility grid. A nonlinear predictive energy management strategy is used for optimal power flow. The energy management strategy uses real-time forecasted load, weather conditions and electricity cost. However, wind turbine and SOFC with appropriate models need to integrate in the system.

To address all the afore mentioned hitches, an efficient Chebyshev wavelet embedded NeuroFuzzy indirect adaptive MPPT control scheme for VSWT-PMSG, an efficient Hermite wavelet embedded NeuroFuzzy indirect adaptive MPPT control scheme for PV system and an effective Hermite wavelet incorporated NeuroFuzzy indirect adaptive control scheme for SOFC system integrated into a grid-connected HPS are proposed. In order to supply uninterrupted and consistent power to the load, a supervisory control policy which consists of nine different modes of operation is also presented. In the stated HPS, tracking the MPP for VSWT-PMSG system, tracking the MPP of PV system and getting the swift response of SOFC are quite difficult, because, this system is highly characterized by nonlinearity. The nonlinearity arises due to the erratic load, variable wind speed, dynamic solar radiation and inconsistent temperature. This work is actually an extension of the work presented in [12], [18], [19]. The rest of the paper is organized into five main sections. Section 2 presents the problem formulation. Section 3 presents the mathematical modeling for VSWT-PMSG, PV and SOFC system. Section 4 gives the details of operation strategy and supervisory control. Simulation results are discussed in section 5. Section 6 concludes the outcomes of this research work.

## 2 Problem formulation

**Fig 1:**
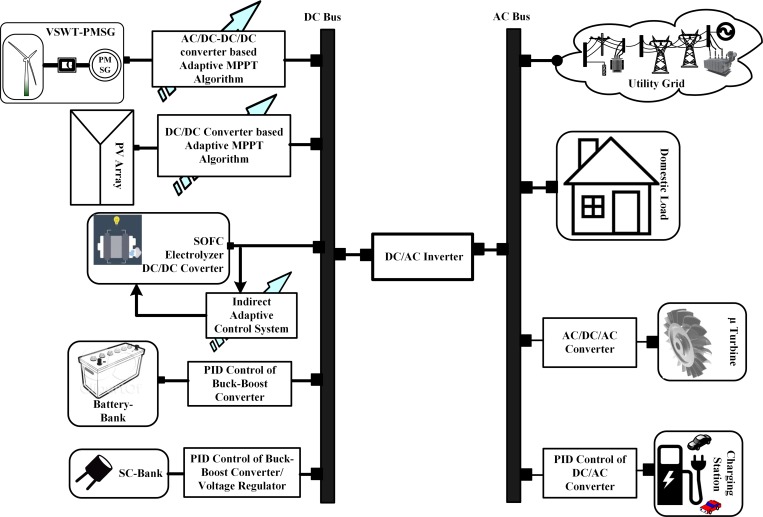
Hybrid power system.

The nonlinear MIMO hybrid power system with renewable energy sources is shown in Fig 1. The auto-regression NeuroFuzzy model of the nonlinear dynamic HPS can be described as:

$$\left[\begin{array}{c} {\hat{y}}_{NF-WT}(k)\\ {\hat{y}}_{NF-PV}(k)\\ {\hat{y}}_{NF-SOFC}(k) \end{array}\right]=\left[\begin{array}{ccc} f_{NF-WT}(\Omega (k)) & 0 & 0\\ 0 & f_{NF-PV}(\Omega (k)) & 0\\ 0 & 0 & f_{NF-SOFC}(\Omega (k)) \end{array}\right]$$ (1)

Where Ω(k) = y(k-1), …, y(k-n), u(k), u(k-1), …, u(k-m). ŷ_NF-WT_(k) gives predictive output at time step *k* for a SISO VSWT system. ŷ_NF-PV_(k) gives predictive output at time step *k* for a SISO PV system. ŷ_NF-SOFC_(k) gives predictive output at time step *k* for a SISO SOFC system. The nonlinear dynamic models for VSWT system, PV system and SOFC system is captured online if:

$$\underset{t\to \infty }{\lim}\Xi_{Ide}=\underset{t\to \infty }{\lim}\left[\begin{array}{c} y_{NF-WT}(k)-{\hat{y}}_{NF-WT}(k)\\ y_{NF-PV}(k)-{\hat{y}}_{NF-PV}(k)\\ y_{NF-SOFC}(k)-{\hat{y}}_{NF-SOFC}(k) \end{array}\right]\Rightarrow \varepsilon_{Ide}$$ (2)

Where ε_Ide_ is the identification error and $\varepsilon_{Ide}=\left[\begin{array}{c} \varepsilon_{1-Ide}\\ \varepsilon_{2-Ide}\\ \varepsilon_{3-Ide} \end{array}\right]$ is a vector of small constant finite values. The hybrid power system is stable if:

$$\underset{t\to \infty }{\lim}\Xi_{Cont}=\underset{t\to \infty }{\lim}\left[\begin{array}{c} y_{NF-WT}(k)-y_{NF-WT-ref}(k)\\ y_{NF-PV}(k)-y_{NF-PV-ref}(k)\\ y_{NF-SOFC}(k)-y_{NF-SOFC-ref}(k) \end{array}\right]\Rightarrow \varepsilon_{Cont}$$ (3)

Where ε_Cont_ is the controller error and $\varepsilon_{Cont}=\left[\begin{array}{c} \varepsilon_{1-Cont}\\ \varepsilon_{2-Cont}\\ \varepsilon_{3-Cont} \end{array}\right]$ is a vector of small constant finite values.

s.t. (general hybrid power system constraints)

$$\{\begin{array}{c} Battery\Rightarrow \{20\%<SOC_{Bat}<90\%\\ SC\Rightarrow \{20\%<SOC_{SC}<90\%\\ \mu Turbine\Rightarrow \{\Delta V_{trm}=\Delta V_{ref}\\ \begin{array}{l} CS\Rightarrow \{\begin{array}{c} 20\%<SOC_{BSS}<90\%\\ 20\%<SOC_{PHEV_{i}}<90\%\;\forall \;i=1,\cdots ,\;5. \end{array}\\ Load\Rightarrow \{\begin{array}{c} THD_{V}<5\%,\;THD_{I}<5\%,\\ -0.8\%<f<+0.8\%\\ -5\%<V_{RMS}<+5\% \end{array}\\ Grid\;stability\Rightarrow \{\begin{array}{c} \Delta P_{DC-bus}=0\\ \begin{array}{l} \Delta P_{AC-bus}=0\\ \Delta Q_{AC-bus}=0 \end{array} \end{array}\\ Main\;inverter\Rightarrow \{\Delta V_{Inv}=\Delta V_{Grid} \end{array} \end{array}$$

The μTurbine, battery storage system, super capacitor and the charging station are interfaced to either DC bus or AC bus via PID-PWM based converters. The DC and AC buses are connected through the main inverter which are controlled by a PID-hysteresis based PWM technique. All these devices are operated by the supervisory control system. They are made to follow their respective reference power signals computed by the supervisory control system. In a guaranteed stable HPS, the change in all the system variables (states) diminishes asymptotically as the time goes to infinity. For the safe and stable operation of the HPS, it is extremely important to supply quality and reliable power to the load. It will also facilitate the smooth and uninterrupted bidirectional flow of power between the grid and HPS.

### 2.1. VSWT-PMSG MPPT subsystem adaptive control design

**Fig 2:**
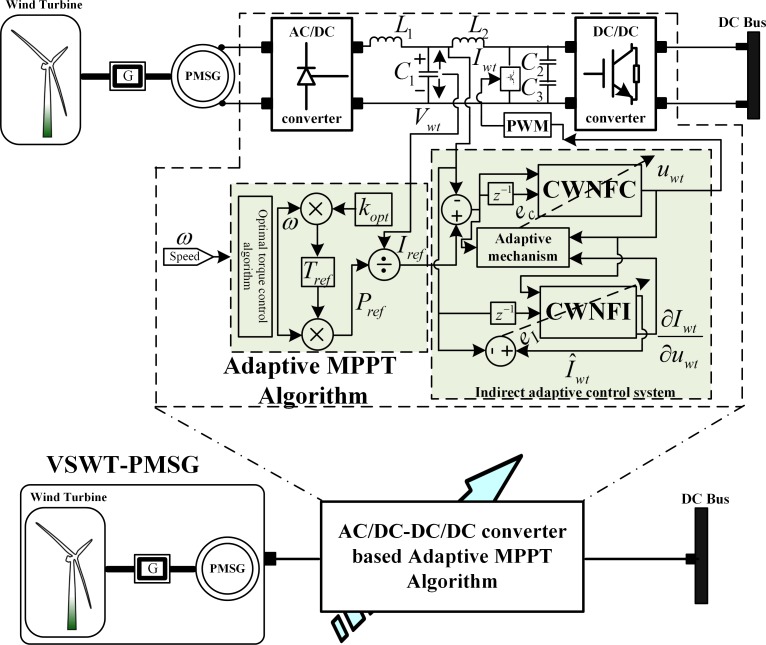
VSWT-PMSG subsystem closed-loop model.

For the wind-turbine subsystem control strategy design, the objective is to acquire the MPP of the wind-turbine subsystem through the indirect adaptive Chebyshev wavelet based NeuroFuzzy algorithm. The unknown subsystem model, *f*_NF-WT_(Ω(K)) is identified online as defined in (1) and depicted in Fig 2.

The nonlinear subsystem dynamic model, *f*_NF-WT_(Ω(K)) for VSWT-PMSG is identified by using the objective function

$$↓\Xi_{WT-Ide}=\frac{1}{2}{\left[I_{NF-WT}\left(k\right)-{\hat{I}}_{NF-WT}(k)\right]}^{2}$$ (4)

s.t. (VSWT-PMSG subsystem model constraints)

$$P_{wind}(k)=\{\begin{array}{l} \;0\;(WT-stall)\;if\;V_{wind}<V_{ci}\;\mathrm{or}\;V_{wind}>V_{co}\\ \eta_{gears}\eta_{gen}N_{WT}C_{p}(\lambda ,\;\beta )\frac{\rho A_{s}}{2}V_{wind\;if\;V_{ci}<V_{wind}<V_{R}}^{3}\\ \;\eta_{gears}\eta_{gen}N_{WT}P_{R}\;if\;V_{R}<V_{wind}<V_{co} \end{array}$$ (5)

Where V_wind_ is the wind speed, V_ci_ is the cut-in wind speed, V_co_ is the cut-out wind speed, *V*_*R*_ is the reference wind speed, η_gears_ is the gearbox efficiency, η_gen_ is the generator efficiency, N_WT_ is the capacity factor, P_R_ is the rated power, A_S_ is the swept area, ρ is the air density, C_p_ is the power coefficient. C_p_ is the function of TSR λ and the blade pitch angle β. Based on Ξ_WT_-_Ide,_ the parameters, ξ_ij_ϵ{m_ij_, σ_ij_, w_ij_} of the Chebyshev wavelet based NeuroFuzzy model, *f*_NF-WT_(Ω(K)) are optimized adaptively. Where m_ij_, σ_ij_ and w_ij_ are the mean, variance and weight of the Gaussian membership function for *ith* input, *jth* rule. Where i = 1, …, n and i = 1, …, m. The update equations for all parameters are:

$$m_{ij}\left(k+1\right)=m_{ij}\left(k\right)+\alpha_{WT}e_{I}\left[\left\{\frac{\beta_{i}-{\hat{I}}_{NF-WT}\left(k\right)}{\sum_{i=1}^{n}\mu_{i}}\right\}\mu_{i}\left\{\frac{x_{i}-m_{ij}}{\sigma_{ij}^{2}}\right\}\right]$$ (6)

$$\sigma_{ij}\left(k+1\right)=\sigma_{ij}\left(k\right)+\alpha_{WT}e_{I}\left[\left\{\frac{\beta_{i}-{\hat{I}}_{NF-WT}\left(k\right)}{\sum_{i=1}^{n}\mu_{i}}\right\}\mu_{i}\left\{\frac{x_{i}-m_{ij}}{\sigma_{ij}^{3}}\right\}\right]$$ (7)

$$w_{ij}\left(k+1\right)=w_{ij}\left(k\right)+\alpha_{WT}e_{I}\left[\left\{\frac{\mu_{i}}{\sum_{i=1}^{n}\mu_{i}}\right\}{ϒ}_{nm}(x_{i})\right]$$ (8)

Where α_WT_ is the learning rate and e_I_ = I_NF-WT_(k)-Î _NF-WT_(k) is the identification error. $\mu_{i}=\prod_{i=1}^{n}\exp\left(\frac{-1}{2}{\left[\frac{x_{i}-m_{ij}}{\sigma_{ij}}\right]}^{2}\right)$, *x*_*i*_ is the *ith* input, β_i_ = wij×Υ_nm_(x_i_) and Υ_nm_(x_i_) is the Chebyshev wavelet which is defined on the interval [0 1] as follows:

$${ϒ}_{nm}(x_{i})=\{\begin{array}{cc} 2^{k/2}\Gamma_{m}(2^{k}x_{i}-2n+1), & \frac{n-1}{2^{k-1}}\leq x_{i}\leq \frac{n}{2^{k-1}}\\ 0, & \mathrm{Otherwise} \end{array}$$ (9)

Where n = 1, 2, …, 2^k-1^ which is the translation parameter and m = 0, 1, …, M-1 is the order of the polynomial.

Γm={1π,m=02πΓ˜m,m>0 (10)

Where ${\tilde{\Gamma }}_{m}$ are Chebyshev polynomials and can be calculated as:

$${\tilde{\Gamma }}_{0}=1,\;{\tilde{\Gamma }}_{1}=x_{i}\;\mathrm{and}\;{\tilde{\Gamma }}_{m+1}=2x_{i}{\tilde{\Gamma }}_{m}-{\tilde{\Gamma }}_{m-1}$$ (11)

The cost function for the controller is:

$$↓\Xi_{WT-Cont}=\frac{1}{2}{\left[I_{NF-WT}\left(k\right)-I_{NF-WT-ref}(k)\right]}^{2}$$ (12)

The control law u_WT_(k) is:

$$u_{WT}(k)=\frac{\sum_{i=1}^{n}\mu_{ij}\beta_{i}}{\sum_{i=1}^{n}\mu_{i}}=\frac{\sum_{i=1}^{n}\left(\left[\prod_{i=1}^{n}\exp\left(\frac{-1}{2}{\left[\frac{x_{i}-m_{ij}}{\sigma_{ij}}\right]}^{2}\right)\right]\times \left[w_{ij}\times {ϒ}_{nm}(x_{i})\right]\right)}{\sum_{i=1}^{n}\left[\prod_{i=1}^{n}\exp\left(\frac{-1}{2}{\left[\frac{x_{i}-m_{ij}}{\sigma_{ij}}\right]}^{2}\right)\right]}$$ (13)

The generalized update equation for control law u_WT_(k) is given as:

$$\chi_{ij}\left(k+1\right)=\chi_{ij}\left(k\right)+\eta_{WT}\frac{\partial {℧}_{WT}\left(k\right)}{\partial \chi_{ij}\left(k\right)}+\eta_{WT}\Delta e_{c}(k+1)$$ (14)

The e_c_ = I_NF-WT_(k)-I_NF-WT-ref_(k) is used to optimize the parameters χ_ij_ϵ{κ_ij_, ϑ_ij_, ν_ij_} of the controller. Where Δ*e*_*c*_(*k* + 1) = *e*_*c*_(*k*)−*e*_*c*_(*k*−1), η_*WT*_ is the learning rate, κ_ij_, ϑ_ij_ and ν_ij_ are the mean, variance and weight of the Gaussian membership function for *ith* input, *jth* rule. The term Ʊ_WT_(k) can be calculated as:

$${℧}_{WT}(k)=\frac{1}{2}\left[e_{c}^{2}(k)+\eta_{WT}u_{WT}^{2}(k)\right]$$ (15)

Where $\frac{\partial {℧}_{WT}(k)}{\partial \chi_{ij}(k)}$ can be simplified as:

$$\frac{\partial {℧}_{WT}(k)}{\partial \chi_{ij}(k)}=\left[e_{c}(k)\frac{\partial {\hat{I}}_{NF-WT}(k)}{\partial u_{WT}(k)}-\eta_{WT}u_{WT}(k)\right]\frac{\partial u_{WT}(k)}{\partial \chi_{ij}(k)}$$ (16)

The term $\frac{\partial {\hat{I}}_{NF-WT}(k)}{\partial u_{WT}(k)}$ can be calculated as:

$$\frac{\partial {\hat{I}}_{NF-WT}(k)}{\partial u_{WT}(k)}=\frac{\sum_{i=1}^{n}\mu_{i}\left[-\left(\frac{u_{WT}(k)-m_{ij}}{\sigma_{ij}^{2}}\right)(\beta_{i}-{\hat{I}}_{NF-WT}(k))+2\sqrt{\frac{2}{\pi }}\left\{8c_{11}^{i}+c_{12}^{i}(128u_{WT}(k)-0.5)\right\}\right]}{\sum_{i=1}^{n}\mu_{i}}$$ (17)

Where $c_{11}^{i}$ and $c_{12}^{i}$ are wavelet coefficients for *ith* input. The MPPT of VSWT-PMSG nonlinear subsystem is guaranteed by the successful convergence of the variables given below through the implementation of the proposed adaptive control algorithm.

$$\underset{t\to \infty }{\lim}\{\begin{array}{c} {\hat{I}}_{NF-WT}\left(k\right)\to I_{NF-WT}\left(k\right)\\ I_{NF-WT}\left(k\right)\to I_{NF-WT-ref}\left(k\right)\\ T_{e}(k)\to T_{m}(k)\\ T_{m}(k)\to T_{ref}(k) \end{array}$$

Where T_e_ is the electrical torque, T_m_ is the mechanical torque and T_ref_ is the reference torque for VSWT-PMSG. The proposed intelligent adaptive control law, u_WT_ will operate the subsystem via AC/DC/DC/DC converter to generate maximum power based on the available wind speed. It ensures the maximum power acquired for a safe wind speed profile (between cut-in and cut-out speeds).

### 2.2. PV MPPT subsystem adaptive control design

**Fig 3:**
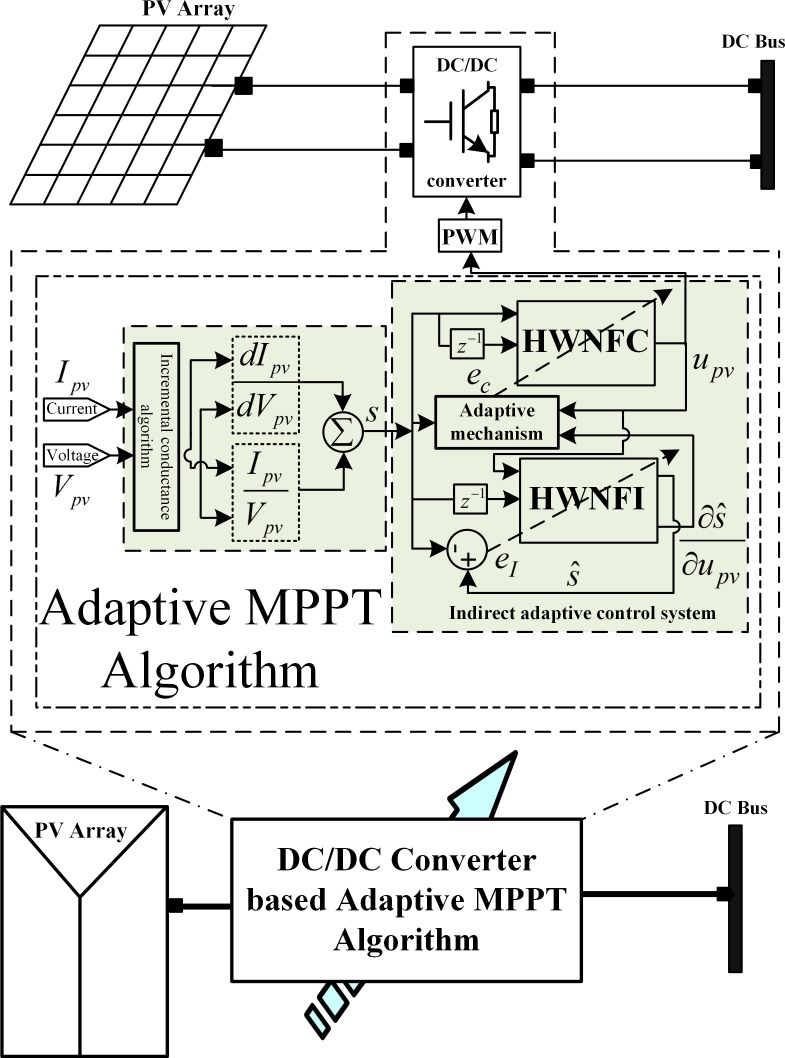
PV subsystem model.

The objective of the photovoltaic subsystem control strategy is to track the maximum operating point through the indirect adaptive Hermite wavelet based NeuroFuzzy algorithm. The unknown subsystem model, *f*_NF-PV_(Ω(K)) is identified online as given in (1) and depicted in Fig 3.

The nonlinear subsystem dynamic model, *f*_NF-PV_(Ω(K)) for PV system is identified by using the objective function

$$↓\Xi_{PV-Ide}=\frac{1}{2}{\left[s\left(k\right)-\hat{s}(k)\right]}^{2}$$ (18)

s.t. (PV subsystem model constraints)

$$\{\begin{array}{c} P_{PV}\leq P_{peak}\\ V_{PV}\leq V_{oc}\\ I_{PV}\leq I_{sc}\\ \varphi_{PV}\leq \varphi_{max}\\ T_{PV}\leq T_{max} \end{array}$$

Where P_peak_ is the peak power, V_oc_ is the open circuit voltage, I_sc_ is the short circuit current, φ_max_ is the maximum solar irradiance and T_max_ is the maximum temperature. They are specified under standard test conditions (STC). When the change of PV power with respect to the operating voltage is zero, then the MPP is achieved as follows:

$${s|}_{MPP}=\frac{\partial P_{pv}}{\partial V_{pv}}{|}_{MPP}={\left[\frac{I_{pv}}{V_{pv}}+\frac{\partial I_{pv}}{\partial V_{pv}}\right]}_{MPP}=0$$ (19)

Where *s* is the slope of PV power with respect to the operating voltage. At MPP, the dynamic input conductance of the subsystem model is equal to the negative of the input static conductance. The change in duty cycle also alters the impedance seen at the input of a DC/DC boost converter. For boost converter, the load impedance (output impedance) is always greater than the input impedance. Based on Ξ_PV_-_Ide_, the parameters, *ζ*_*ij*_*ϵ*{ƛ_*ij*_,*γ*_*ij*_,*ϖ*_*ij*_} of the Hermite wavelet based NeuroFuzzy model, *f*_NF-PV_(Ω(K)) are optimized adaptively. The Hermite wavelet ψ_a,b_(x_i_) is defined on the interval [0, 1] as follows:

$$\psi_{a,b}(x_{i})=\{\begin{array}{cc} 2^{c/2}\sqrt{\frac{1}{a!2^{a}\sqrt{\pi }}}H_{b}(2^{c}x_{i}-\overset{⌢}{a}), & \frac{\overset{⌢}{a}-1}{2^{c}}\leq x_{i}\leq \frac{\overset{⌢}{a}-1}{2^{c}}\\ 0, & \mathrm{Otherwise} \end{array}$$ (20)

Where x_i_ is the *ith* input, c = 1, 2, …, c_n_ is the level of resolution, a = 1, 2, …, 2^c-1^, â = 2a-1 is the translation parameter, and b = 1, 2, …, B-1 is the order of the polynomial, B>0. Where H_b_ is orthogonal pertaining to the weight function as:

$$\int_{-\infty }^{\infty }e^{-x^{2}}H_{b}H_{a}=\{\begin{array}{cc} 0, & b\neq a\\ a!2^{a}\sqrt{\pi }, & b=a \end{array}$$ (21)

The Hermite polynomial H_b_ of order b is defined on the interval [-∞,∞] and is given as

$$H_{0}=1,\;H_{1}=2x_{i}\;\mathrm{and}\;H_{b+1}=2x_{i}H_{b}-2bH_{b-1}$$ (22)

The update equations for all parameters are:

$${ƛ}_{ij}\left(k+1\right)={ƛ}_{ij}\left(k\right)+\alpha_{PV}e_{I-PV}\left[\left\{\frac{{\bar{\beta }}_{i}-\hat{s}\left(k\right)}{\sum_{i=1}^{n}{\bar{\mu }}_{i}}\right\}{\bar{\mu }}_{i}\left\{\frac{x_{i}-{ƛ}_{ij}}{\gamma_{ij}^{2}}\right\}\right]$$ (23)

$$\gamma_{ij}\left(k+1\right)=\gamma_{ij}\left(k\right)+\alpha_{PV}e_{I-PV}\left[\left\{\frac{{\bar{\beta }}_{i}-\hat{s}\left(k\right)}{\sum_{i=1}^{n}{\bar{\mu }}_{i}}\right\}{\bar{\mu }}_{i}\left\{\frac{x_{i}-{ƛ}_{ij}}{\gamma_{ij}^{3}}\right\}\right]$$ (24)

$$\varpi_{ij}\left(k+1\right)=\varpi_{ij}\left(k\right)+\alpha_{PV}e_{I-PV}\left[\left\{\frac{{\bar{\mu }}_{i}}{\sum_{i=1}^{n}{\bar{\mu }}_{i}}\right\}\psi_{ab}(x_{i})\right]$$ (25)

Where α_PV_ is the learning rate and $e_{I-PV}=s\left(k\right)-\hat{s}(k)$ is the identification error. ƛ_*ij*_, *γ*_*ij*_ and *ϖ*_*ij*_ are the mean, variance and weight of Gaussian membership function for *ith* input, *jth* rule, i.e., ${\bar{\mu }}_{i}=\prod_{i=1}^{n}\exp\left(\frac{-1}{2}{\left[\frac{x_{i}-{ƛ}_{ij}}{\gamma_{\partial ij}}\right]}^{2}\right)$ and ${\bar{\beta }}_{i}=\varpi_{ij}\times \psi_{ab}(x_{i})$.

The cost function for the controller is:

$$↓\Xi_{PV-Cont}=\frac{1}{2}{\left[s\left(k\right)-r(k)\right]}^{2}$$ (26)

The control law u_pv_(k) is:

$$u_{PV}(k)=\frac{\sum_{i=1}^{n}{\bar{\mu }}_{ij}{\bar{\beta }}_{i}}{\sum_{i=1}^{n}{\bar{\mu }}_{i}}=\frac{\sum_{i=1}^{n}\left(\left[\prod_{i=1}^{n}\exp\left(\frac{-1}{2}{\left[\frac{x_{i}-{ƛ}_{ij}}{\gamma_{\partial ij}}\right]}^{2}\right)\right]\times \left[\varpi_{ij}\times \psi_{ab}(x_{i})\right]\right)}{\sum_{i=1}^{n}\left[\prod_{i=1}^{n}\exp\left(\frac{-1}{2}{\left[\frac{x_{i}-{ƛ}_{ij}}{\gamma_{\partial ij}}\right]}^{2}\right)\right]}$$ (27)

The e_c-PV_ = s(k)-r(k) is used to optimize the parameters ℵ_ij_ϵ{℘_ij_, ϛ_ij_, τ_ij_} of control law u_pv_(k)_**.**_ The generalized update equation is given as follows:

$${ℵ}_{ij}\left(k+1\right)={ℵ}_{ij}\left(k\right)+\eta_{PV}\frac{\partial {℧}_{PV}\left(k\right)}{\partial {ℵ}_{ij}\left(k\right)}+\eta_{PV}\Delta e_{c-PV}(k+1)$$ (28)

Where Δe_c_-_PV_(k+1) = Δe_c_-_PV_(k)- Δe_c_-_PV_(k-1), η_PV_ is the learning rate, ℘_ij_, ϛ_ij_, and τ_ij_ are the mean, variance and weight of the Gaussian membership function for *ith* input, *jth* rule. The term Ʊ_PV_ is calculated as:

$${℧}_{PV}(k)=\frac{1}{2}\left[e_{c-PV}^{2}(k)+\eta_{PV}u_{PV}^{2}(k)\right]$$ (29)

Where $\frac{\partial {℧}_{PV}(k)}{\partial {ℵ}_{ij}(k)}$ can be simplified using the following equation:

$$\frac{\partial {℧}_{PV}(k)}{\partial {ℵ}_{ij}(k)}=\left[e_{c-PV}(k)\frac{\partial \hat{s}(k)}{\partial u_{PV}(k)}-\eta_{PV}u_{PV}(k)\right]\frac{\partial u_{PV}(k)}{\partial {ℵ}_{ij}(k)}$$ (30)

The term $\frac{\partial \hat{s}(k)}{\partial u_{PV}(k)}$ can be calculated as follows:

$$\frac{\partial \hat{s}(k)}{\partial u_{PV}(k)}=\frac{\sum_{i=1}^{n}{\bar{\mu }}_{i}\left[-\left(\frac{u_{pv}-\hbar_{ij}}{\gamma_{ij}^{2}}\right)({\bar{\beta }}_{i}-\hat{s}(k))+2\sqrt{\frac{2}{\pi }}\left\{8q_{11}^{i}+q_{12}^{i}(128u_{PV}(k)-L)\right\}\right]}{\sum_{i=1}^{n}{\bar{\mu }}_{i}}$$ (31)

Where $q_{11}^{i}$ and $q_{12}^{i}$ are Hermite wavelet coefficients.

$$\begin{array}{cc} L=32, & 0\leq u_{PV}(k)\leq 1/2\\ L=48, & 1/2\leq u_{PV}(k)\leq 1 \end{array}\}$$ (32)

The MPPT of PV subsystem is accomplished by the successful convergence of the variables given below through the implementation of the proposed adaptive control algorithm.

$$\underset{t\to \infty }{\lim}\{\begin{array}{c} \hat{s}\left(k\right)\to s\left(k\right)\\ s\left(k\right)\to 0\\ V_{PV}(k)\to V_{PV-MPP}(k)\\ P_{PV}(k)\to P_{PV-MPP}(k) \end{array}$$

The proposed intelligent adaptive control law, u_PV_ will operate the subsystem via DC/DC converter to extract maximum power according to the irradiance and temperature. The maximum power extraction is guaranteed under the unpredictable climatic conditions.

### 2.3 SOFC adaptive control problem

The SOFCs have sluggish dynamic response as compared to the dynamic responses of the power conditioner and the load. The SOFC incapability to alter its electrical output, i.e., current as swiftly as the electrical load variation has significant consequences on the overall HPS. Therefore, the dynamic response of SOFC has substantial importance. For the SOFC swift response, the quantity of input hydrogen flow rate must be controlled. The input hydrogen flow rate is proportional to the SOFC stack current. Therefore, the optimal flow rate of input hydrogen is achieved by controlling the SOFC stack current. The relationship for the SOFC stack current is given by:

$$m_{H_{2}}^{in}=\left(\frac{2l}{H_{2}^{uti}}\right)I_{SOFC}\Rightarrow I_{SOFC}=\left(\frac{H_{2}^{uti}}{2l}\right)m_{H_{2}}^{in}$$ (33)

Where $H_{2}^{uti}$ is the optimal hydrogen utilization, and $m_{H_{2}}^{in}$ is the molar flow of input hydrogen. $H_{2}^{uti}$ has a typical range of 80–90%. For optimal hydrogen utilization, the SOFC current lies within the following limits:

$$\frac{0.8m_{H_{2}}^{in}}{2l}=I_{SOFC-\min}\leq I_{SOFC-ref}\leq I_{SOFC-\max}=\frac{0.9m_{H_{2}}^{in}}{2l}$$ (34)

Where l is the constant that gives the amount of hydrogen reacting in the SOFC, and $0.8m_{H_{2}}^{in}$ and $0.9m_{H_{2}}^{in}$ are the minimum and maximum limits of molar flow of hydrogen, respectively. I_SOFC-min_ I_SOFC-ref_ and I_SOFC-max_ are the minimum reference and maximum SOFC currents, respectively. The SOFC power demand is converted into the current as follows:

$$I_{SOFC-ref}=\frac{P_{SOFC-ref}}{V_{SOFC}}$$ (35)

**Fig 4:**
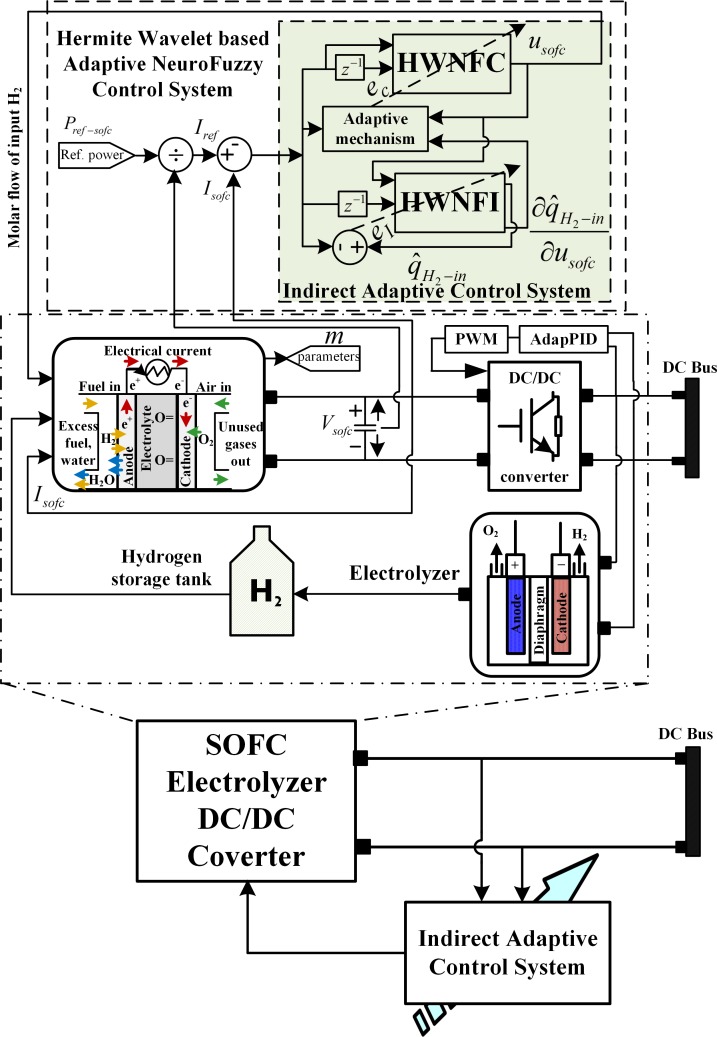
SOFC-Electrolyzer subsystem model.

The unknown subsystem model, *f*_NF-SOFC_(Ω(K)) is identified online as defined in (1). The SOFC- electrolyzer subsystem along with hydrogen storage tank is depicted by Fig 4. The nonlinear subsystem dynamic model, *f*_NF-SOFC_(Ω(K)) for SOFC is identified by using the objective function

$$↓\Xi_{SOFC-Ide}=\frac{1}{2}{\left[I_{NF-SOFC}\left(k\right)-{\hat{I}}_{NF-SOFC}(k)\right]}^{2}$$ (36)

s.t.

1. (SOFC subsystem model constraints)
  $$\{\begin{array}{c} Fuel\;flow=con\tan t\\ Fuel\;utilization=con\tan t\\ Fuel\;starvation≅0\\ P_{SOFC}(k)\leq P_{Peak}(k) \end{array}$$
2. (Electrolyzer constraints)
  $$\{V_{Elect}\leq V_{DC-bus}$$

Based on the function, Ξ_SOFC_-_Ide,_ minimization, the parameters, *ζ*_*ij*_*ϵ*{ƛ_*ij*_,*γ*_*ij*_,*ϖ*_*ij*_} of the Hermite- wavelet based NeuroFuzzy model, *f*_NF-SOFC_(Ω(K)) are optimized adaptively using the update Eqs (23–25).

The SOFC nonlinear subsystem convergence is guaranteed if the following variables converge successfully using the proposed adaptive control algorithm.

$$\underset{t\to \infty }{\lim}\{\begin{array}{c} {\hat{I}}_{NF-SOFC}\left(k\right)\to I_{NF-SOFC}\left(k\right)\\ I_{NF-SOFC}\left(k\right)\to I_{NF-SOFC-ref}\left(k\right)\\ H_{2}\left(k\right)\to H_{2-ref}\left(k\right) \end{array}$$

The proposed intelligent adaptive control law, u_SOFC_(k) will utilize the subsystem via DC/DC converter to deliver hydrogen swiftly and consequently the output current, I_NF-SOFC_(k). The control law u_SOFC_(k) is:

$$u_{SOFC}(k)=\frac{\sum_{i=1}^{n}{\bar{\mu }}_{ij}{\bar{\beta }}_{i}}{\sum_{i=1}^{n}{\bar{\mu }}_{i}}=\frac{\sum_{i=1}^{n}\left(\left[\prod_{i=1}^{n}\exp\left(\frac{-1}{2}{\left[\frac{x_{i}-{ƛ}_{ij}}{\gamma_{\partial ij}}\right]}^{2}\right)\right]\times \left[\varpi_{ij}\times \psi_{ab}(x_{i})\right]\right)}{\sum_{i=1}^{n}\left[\prod_{i=1}^{n}\exp\left(\frac{-1}{2}{\left[\frac{x_{i}-{ƛ}_{ij}}{\gamma_{\partial ij}}\right]}^{2}\right)\right]}$$ (37)

## 3 Mathematical modeling

### 3.1 Variable Speed Wind Energy Conversion System (VS-WECS) modeling

#### 3.1.1 Wind turbine model for maximum power extraction

The aero dynamic power P_a_ caught by the wind turbine is:

$$P_{a}=0.5\rho R^{2}C_{p}(\lambda )V_{wind}^{3}$$ (38)

Where, V_co_> V_wind_ > V_ci_. λ is the ratio of the tip speed of the turbine blades to the wind speed is $\lambda =\frac{R\omega_{r}}{V_{wind}}$.

The rotor power (aerodynamic power) is also defined by:

$$P_{a}=\omega_{r}T_{a}$$ (39)

The aerodynamic torque T_a_ is:

$$T_{a}=\frac{1}{2}\pi \rho R^{3}C_{q}(\lambda )V_{wind}^{2}$$ (40)

The T_a_ drives the wind turbine at the ω_r_ (speed). For a specific wind speed, the output power is proportional to the rotor speed, i.e., P_a_ = kω_r_^2^. Where $k=0.5\rho AC_{p}{\left(\frac{R}{\lambda }\right)}^{3}$ and the optimum aerodynamic rotor power is captured by controlling the rotor speed ω_r_. For a certain wind speed, the optimum power is:

$$P_{a,opt}=k_{opt}\omega_{r}^{3}$$ (41)

Where $k_{opt}=0.5\rho AC_{p,opt}{\left(\frac{R}{\lambda_{opt}}\right)}^{3}$.

The optimum power acquisition from the wind refers to acquiring the necessary power under fluctuating wind speed condition.

### 3.2 Photovoltaic Energy Conversion System (PVECS) modeling

#### 3.2.1 Modeling of photovoltaic subsystem

A photovoltaic cell converts photon energy directly into electrical energy in the form of direct current which means that a photovoltaic device model must be based on the electrical characteristics. The average photovoltaic array model consists of two controlled current sources I_pv-1_ and I_pv-2_. The current source I_pv-1_ is controlled by I_L_.

$$I_{L}=\frac{s}{s_{ref}}\left(I_{L-ref}+\alpha_{isc}\left(T_{cell-K}-T_{ref-K}\right)\right)$$ (42)

The controlled current source I_pv-2_ depends upon I_L2_.

$$I_{L2}=I_{d}+I_{Rsh}$$ (43)

$$I_{d}=I_{0}\left[\exp\left(v_{d}-v_{T}\right)-1\right]$$ (44)

vT=vTref(Tcell−KTref−K) (45)

I0=I0−ref[(Tcell−KTref−K)3exp(EgrefK1Tref−K)−(E−gK1Tcell−K)] (46)

$$E_{g}=E_{gref}\left(1+dE_{g}dT\left(T_{cell-K}-T_{ref-K}\right)\right)$$ (47)

IRsh=vd(RshNserNpar)ss−ref (48)

Where s = irradiance, I_d_ = diode current, v_d_ = diode voltage, I_0_ = diode saturation current, v_T_ = temperature voltage = $\frac{kT_{cell-K}}{qnIN_{cell}N_{ser}}$, T_cell-K_ = cell temperature, k = Boltzman constant = 1.3806×10^−23^ *Jk*^−1^, q = electron constant = 1.6022×10^−19^ *C*, nI = diode identity factor, N_cell_ = no. of series connected cells per module, N_ser_ = no. of series connected modules per string, N_par_ = no. of parallel connected modules per string. The output voltage of the PV array V_PV_ is given as:

$$V_{PV}=v_{d}-R_{s-array}I_{PV}$$ (49)

Where v_d_ is the voltage across R_sh_ and $R_{s-array}=R_{s}\frac{N_{ser}}{N_{par}}$. The output power of the PV array at the PV terminals is given as:

$$P_{PV}=V_{PV}I_{PV}$$ (50)

### Solid Oxide Fuel Cell Energy Conversion System (SOFCECS) modeling

#### 3.3.1 Modeling of SOFC subsystem

**Fig 5:**
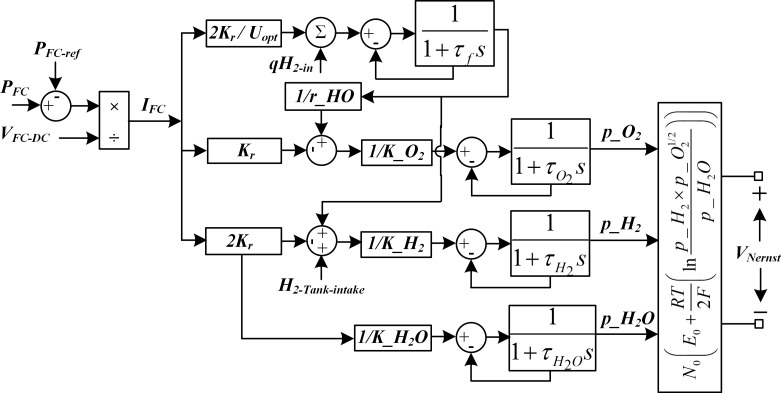
SOFC system dynamic model.

The SOFC dynamic model is shown in Fig 5. The molar flow of hydrogen H_2_ through the valve is proportional to its partial pressure, and is given as:

$$\frac{q_{H_{2}}}{p_{H_{2}}}=\frac{k}{\sqrt{m_{H_{2}}}}=K\_H_{2}$$ (51)

Where k is the anode valve constant, $m_{H_{2}}$ is the molecular mass of hydrogen, $q_{H_{2}}$ is the molar flow of hydrogen, $p_{H_{2}}$ is the partial pressure of hydrogen and K_H_2_ is the valve molar constant of hydrogen. The molar flow of hydrogen that reacts is calculated as:

$$q_{H_{2}}^{r}=\frac{N_{0}I_{SOFC}}{2F}=2K_{r}I_{SOFC}$$ (52)

Where N_0_ is the number of series cells in the stack, F is the Faraday’s constant, K_r_ is the constant and I_SOFC_ is the stack current. The partial pressure of hydrogen P_H_2_ yields the following expression:

p_H2=1K_H21+τH2s(qH2in−2KrISOFC) (53)

Where,

$$\tau_{H_{2}}=\frac{v_{anode}}{K\_H_{2}\times RT}$$ (54)

The v_anode_ represents the volume of the anode, R is the universal gas constant and T is the absolute temperature. A similar operation is made to calculate the partial pressures of oxygen and water. The Nernst’s equation used to calculate the stack output voltage is represented as:

$$V_{Nernst}=E_{0}+\frac{RT}{2F}\ln\left\{\frac{p\_H_{2}\times p\_O_{2}^{1/2}}{p\_H_{2}O}\right\}$$ (55)

Where E_0_ is the voltage associated with the reaction free energy, p_O_2_ and p_H_2_O are the partial pressures of oxygen and water. The output voltage of SOFC is given as:

$$V_{SOFC}=V_{Nernst}-V_{A}-V_{O}-V_{C}$$ (56)

where V_SOFC_ is the SOFC output voltage, V_Nernst_ is the Nernst potential, V_A_ is the activation polarization, V_0_ is the ohmic polarization, and V_C_ is the concentration polarization.

## 4. Operation strategy and supervisory control

### 4.1. General description of the operation strategy

The prime obligation of the supervisory control is to ensure continuous and reliable power supply to the load. The stated HPS has two types of dynamic loads, one is residential load and other is charging station load. The total load is the accumulative of both, i.e., load = residential load + charging station load. There are nine different modes of operation and the supervisory control switches between these modes depending upon the operation strategy. These operation modes are determined by the energy balance between the total generation and the total load. The Modes 1–5 deal with the deficient power, i.e., generation is less than the load, whereas, Modes 6–9 deal with the excess power, i.e., generation is greater than the load.

### 4.2. Supervisory control policy

The following control actions take place in every mode of operation.

***Mode 1*:** If $P_{D\_ref}\geq P_{Ren}\;\&\;SOC_{Bat}\geq 20\%\Rightarrow \left\{ \begin{array}{l} P_{Ren}=P_{WT-\max}+P_{PV-\max}\\ P_{Bat}=P_{Bat-d} \end{array}\right.$

Where *P*_*D_ref*_ = *P*_*Load*_ = *P*_*r*e*sidential*_ + *P*_*CS*_ = *I*_*L*_ × *V*_*L*_, SOC_Bat_ is the state-of-charge of battery and P_Bat-d_ represents the battery in discharge mode. This mode of operation is characterized by both generation subsystems, i.e., WT and PV set to operate at their maximum energy conversion points but the load is not met by the maximum power of WT and PV. The battery is in discharge mode to meet the load. During this mode, only renewable energy sources and battery system deliver the power to the load.

***Mode 2*:** If $P_{D\_ref}\geq P_{Ren}\;\&\;SOC_{Bat}\geq 20\%\;\&\;SOC_{SC}\geq 20\%\Rightarrow \left\{ \begin{array}{l} P_{Ren}=P_{WT-\max}+P_{PV-\max}\\ P_{Bat}=P_{Bat-d}\\ P_{SC}=P_{SC-d} \end{array}\right.$

Where SOC_SC_ is the state-of-charge of SC. In this mode of operation, WT and PV subsystems deliver their maximum power but the load still requires the power. The battery is in discharge mode which means the battery is delivering power to the load. The remaining deficient power is delivered by SC. The SOFC and MT are kept OFF and also, there is no need to take the power from the utility grid, because, the load is met by power taken from renewable sources and storage system.

***Mode 3*:** If $P_{D\_ref}\geq P_{Ren}\;\&\;SOC_{Bat}\geq 20\%\;\&\;SOC_{SC}\geq 20\%\Rightarrow \left\{ \begin{array}{l} P_{Ren}=P_{WT-\max}+P_{PV-\max}\\ P_{Bat}=P_{Bat-d}\\ P_{SC}=P_{SC-d}\\ P_{SOFC}=P_{SOFC-r} \end{array}\right.$

Where P_SOFC_ is the SOFC power. During this mode of operation, the load is met by the cumulative power taken from WT, PV, battery, SC and SOFC. The battery and SC both are in discharge mode but the load requires more power. The SOFC is set to track a power reference. This reference corresponds to the power required to satisfy the total power demand.

***Mode 4*:** If $P_{D\_ref}\geq P_{Ren}\;\&\;SOC_{Bat}\geq 20\%\;\&\;SOC_{SC}\geq 20\%\Rightarrow \left\{ \begin{array}{l} P_{Ren}=P_{WT-\max}+P_{PV-\max}\\ P_{Bat}=P_{Bat-d}\\ P_{SC}=P_{SC-d}\\ P_{SOFC}=P_{SOFC-r}\\ P_{Grid}=P_{Grid-r} \end{array}\right.$

Where P_Grid_ represents the grid power. In this mode, WT, PV, battery, SC and SOFC deliver the power to the load but the load requires more power which is given by the utility grid.

***Mode 5*:** If $P_{D\_ref}\geq P_{Ren}\;\&\;SOC_{Bat}\geq 20\%\;\&\;SOC_{SC}\geq 20\%\Rightarrow \left\{ \begin{array}{l} P_{Ren}=P_{WT-\max}+P_{PV-\max}\\ P_{Bat}=P_{Bat-d}\\ P_{SC}=P_{SC-d}\\ P_{SOFC}=P_{SOFC-r}\\ P_{Grid}=0\\ P_{MT}=P_{MT-r} \end{array}\right.$

Where P_MT_ represents the micro-turbine power. In this mode of operation, the utility grid is unavailable due to peak demand hours. The WT and PV subsystem deliver their maximum powers to the load. The battery and SC both are in the discharge mode, SOFC also delivers the power to the load. The MT supplies the remaining deficient power to the load.

***Mode 6*:** If $P_{D\_ref}\leq P_{Ren}\;\&\;SOC_{Bat}\geq 20\%\Rightarrow \left\{ \begin{array}{l} P_{Exss}=P_{D-ref}-P_{Ren}\\ P_{Bat}=P_{Bat-d}\\ P_{Elect}=P_{Exss} \end{array}\right.$

Where P_Elect_ represents the electrolyzer power. In this mode of operation, there is some excess power P_Exss_ in the system. This excess power is utilized by electrolyzer. The battery remains in the discharge mode.

***Mode 7*:** If $P_{D\_ref}\leq P_{Ren}\;\&\;SOC_{Bat}\geq 20\%\;\&\;SOC_{SC}\leq 90\%\Rightarrow \left\{ \begin{array}{l} P_{Bat}=P_{Bat-d}\\ P_{SC-c}=P_{Exss}\\ P_{Elect}=P_{Exss}-P_{SC-c} \end{array}\right.$

Where P_SC-r_ represents the SC is in charge mode. During this mode, the renewable energy sources generate more power than load. The battery is in discharge mode but the SOC of SC is less than 90%, so the excess power is utilized to charge SC and then by the electrolyzer.

***Mode 8*:** If $P_{D\_ref}\leq P_{Ren}\;\&\;SOC_{Bat}\leq 20\%\;\&\;SOC_{SC}\geq 90\%\Rightarrow \left\{ \begin{array}{l} P_{Bat}=P_{Bat-d}\\ P_{SC-c}=P_{Exss}\\ P_{Grid-r}=P_{Exss}-P_{SC-c}\\ P_{Elect}=P_{Exss}-P_{SC-c}-P_{Grid-r} \end{array}\right.$

Where P_Grid_ is the electrolyzer power. In this mode, the WT and PV generate more power than load. The SC is in charge mode. The excess power after charging the SC is given to the utility grid. There is still some excess power which is utilized by electrolyzer to produce hydrogen for SOFC.

***Mode 9*:** If $P_{D\_ref}\leq P_{Ren}\;\&\;SOC_{SC}\geq 90\%\Rightarrow \left\{ \begin{array}{l} P_{Bat}=0\\ P_{SC-c}=P_{Exss}\\ P_{Grid-r}=P_{Exss}-P_{SC-c}\\ P_{Elect}=P_{Exss}-P_{SC-c}-P_{Grid-r} \end{array}\right.$

In this mode of operation, the battery is disconnected, i.e., neither it will charge nor discharge. The generation is greater than load. The SC is in charge mode. The excess power is given to utility and then to the electrolyzer.

## 5 Results and discussion

The performance of the stated HPS and proposed controllers is evaluated in MATLAB/Simulink R2015a. In an 11 kV grid-connected HPS, wind generation of 100 kW, PV of 260 kW, SOFC of 200 kW, electrolyzer of 150 kW, and MT of 200 kVA along with backup sources (200 Ah battery and 165 F Super-Capacitor) are modeled for the accumulative dynamic residential and charging station load. Defense Housing Authority (DHA), Islamabad, Pakistan, is taken as a case study. The hourly basis wind speed (m/s), irradiance (W/m^2^) and ambient temperature (°C) levels are recorded by the Pakistan Meteorological Department (PMD). The stated HPS with suggested controllers is tested for the 24-hrs period of 22nd June 2015. In this study, a Chebyshev wavelet incorporated NeuroFuzzy indirect adaptive controller (CWNFC) is used to extract the maximum power from WT subsystem. A Hermite wavelet embedded NeuroFuzzy indirect adaptive controller (HWNFC) is used to acquire the maximum power from PV subsystem. A Hermite wavelet incorporated NeuroFuzzy indirect adaptive controller (HWNFC) is used to get the swift response from SOFC subsystem. To evaluate the performance of CWNFC, an adaptive indirect NeuroFuzzy Takagi-Sugeno-Kang controller (ITSKC), an adaptive direct NeuroFuzzy TSK controller (TSKC) and a hysteresis based PI controller (Hyst-PI) are also used to extract the MPP from WT subsystem. For PV subsystem, the performance of HWNFC is compared with ITSKC, TSKC and incremental conductance based PI controller (InCond-PI). The swift response from SOFC subsystem with HWNFC is also compared with ITSKC, TSKC and conventional PI controller.

**Fig 6:**
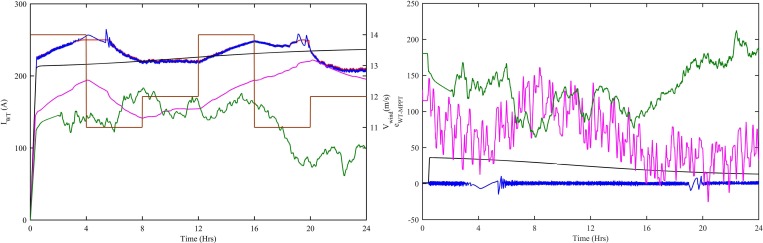
WT (a) MPP currents and wind speed (b) MPPT errors with all four controllers. Ref. is designated as red, CWNFC as blue, ITSKC as black, TSKC Magenta and Hyst-PI as green.

The MPP current of the WT subsystem is shown in Fig 6 (A). The variable speed wind-turbine is used. The base wind speed is taken as 12 m/s, whereas, wind speed varies between 11 and 14 m/s as shown in Fig 6 (A). For t = 0–4 hrs, wind speed is 14 m/s. During this time period, the current of WT subsystem also increases to 256.4 A. From 4–8 hrs, wind speed drops to 11 m/s and WT current also starts decreasing and reaches 220A at t = 8 hrs. From 8–12 hrs, wind speed again increases to 12 m/s and keeps increasing to 14 m/s till t = 16 hrs. The WT current also changes according to the wind speed, i.e., starts increasing from 8–12 hrs and reaches 226 A at t = 12 hrs and keeps increasing to 249.5 A at t = 16 hrs. In next time interval, i.e., t = 16–20 hrs, again wind speed decreases from 14 m/s to 11 m/s and the WT current also starts decreasing and reaches to 242.8 A at t = 18.53 hrs. In 20–24 hrs, a small change in wind speed, i.e., from 11 m/s to 12 m/s also alters the WT current accordingly. As the WT dynamics cannot change abruptly with the wind speed, therefore, the effect of the change in current is slow. The CWNF, ITSKC, TSKC and Hyst-PI all extract the power from WT. The power extraction using CWNFC is more precise, because, the percentage error using CWNFC is 3.2%, ITSKC is 6.4%, TSKC is 12.4% and Hyst-PI is 42%. The WT MPPT errors with CWNF, ITSKC, TSKC and Hyst-PI are shown in Fig 6 (B). The CWNFC has the minimum error as compared to ITSKC, TSKC and Hyst-PI.

**Fig 7:**
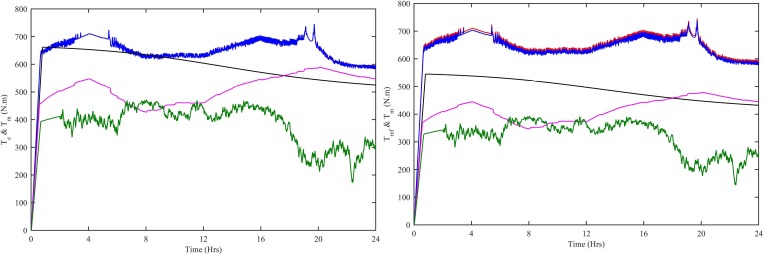
WT (a) Electrical vs mechanical torque (b) Mechanical vs Reference torque. Ref. is designated as red, CWNFC as blue, ITSKC as black, TSKC Magenta and Hyst-PI as green.

The WT subsystem has two types of torques, one is T_m_ produced by wind-turbine and the other is T_e_ produced by PMSG. The variation in wind speed alters the T_m_ of wind-turbine. The T_e_ of PMSG changes according to the T_m_ of wind-turbine. Fig 7 (A) shows the change in T_e_ with respect to T_m_. So, T_m_ becomes the reference for T_e_. Fig 7 (B) shows the change in T_m_ according to reference torque. All the four controllers, i.e., CWNFC, ITSKC, TSKC and Hyst-PI capture both the torques but the CWNFC acquires the torques with the minimum steady error.

**Fig 8:**
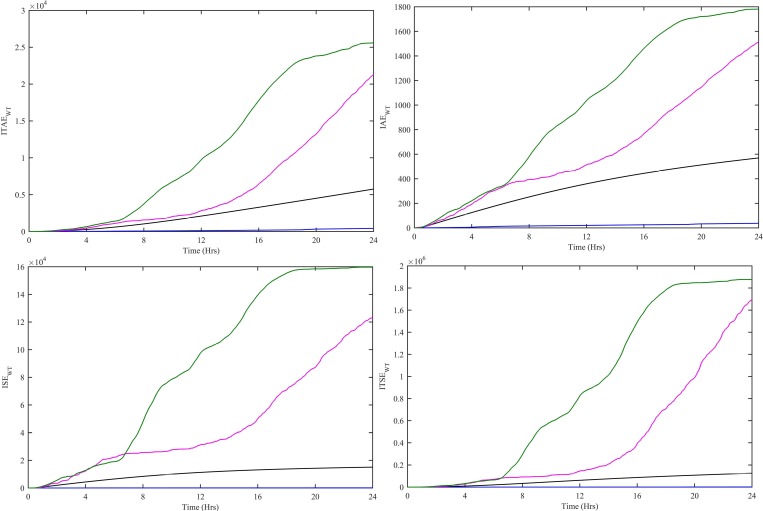
Performance indexes (a) ITAE, (b) IAE, (c) ISE and (d) ITSE for WT controllers. CWNFC is designated as blue, ITSKC as black, TSKC Magenta and Hyst-PI as green.

In order to evaluate the performance of all four controllers, various performance indexes, i.e., Integral Time-weighted Absolute Error (ITAE), Integral Absolute Error (IAE), Integral Squared Error (ISE) and Integral Time-weighted Squared Error (ITSE) are computed. These performance indexes are computed using e_WT_(t) = P_WT-ref_(t)-P_WT_(t) for CWNFC, ITSKC, TSKC and Hyst-PI. The CWNFC indexes have the least and flattest profile as shown in Fig 8. The CWNFC can track with a more stable output WT power as compared to other controllers.

**Fig 9:**
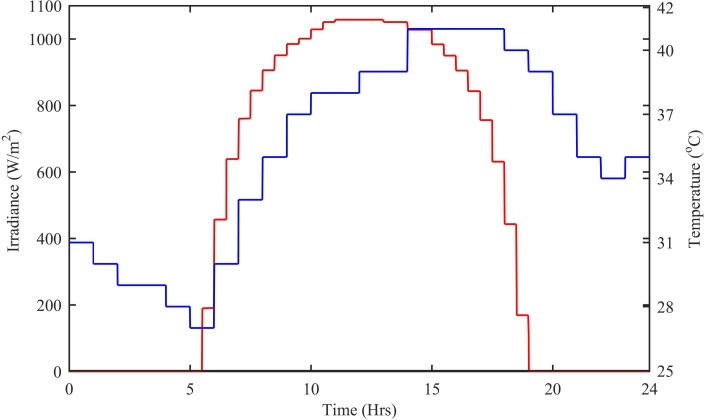
Irradiance and temperature levels for 22^nd^ June 2015. Irradiance is designated as red and temperature as blue.

The output power of the PV subsystem depends upon the ambient temperature and irradiance level which are shown in Fig 9. For t = 0–6 hrs, when the sun power is not available, the ambient temperature also decreases and reaches 27°C at t = 6 hrs. During 5.5–12 hrs, the irradiance level keeps increasing and reaches to 1058 W/m^2^. After t = 12 hrs, the irradiance level starts decreasing and become zero at t = 19 hrs. The ambient temperature reaches a maximum value of 41°C at t = 15 hrs, but after 15 hrs, the temperature starts decreasing again.

**Fig 10:**
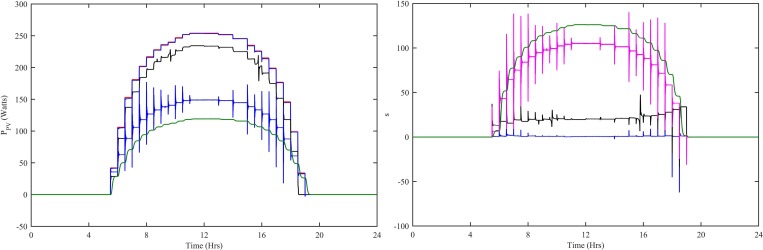
PV (a) MPP power (b) MPPT error with all four controllers. Ref. is designated as red, HWNFC as blue, ITSKC as black, TSKC Magenta and InCond-PI as green.

The maximum powers extracted by PV subsystem using HWNFC, ITSKC, TSKC and InCond-PI are shown Fig 10 (A). The HWNFC extracts the PV subsystem output power with steady-state error = 0.4 kW, % overshoot = 0 and % undershoot = -2. The ITSKC extracts the PV subsystem output power with steady-state error = 20.03 kW, % overshoot = 6 and % undershoot = -13. The TSKC extracts the PV subsystem output power with steady-state error = 105 kW, % overshoot = 61 and % undershoot = -92. The InCond-PI extracts the PV subsystem output power with steady-state error = 135 kW, % overshoot = 2 and % undershoot = -8. The HWNFC has minimum steady-state error, % overshoot and % undershoot as shown in Fig 10 (B). HWNFC has lowest values of all the indexes.

**Fig 11:**
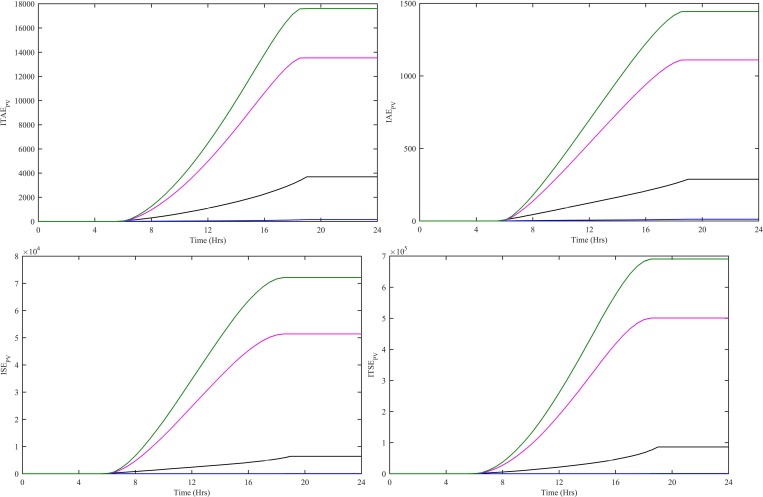
Performance indexes (a) ITAE, (b) IAE, (c) ISE and (d) ITSE for PV controllers. HWNFC is designated as blue, ITSKC as black, TSKC Magenta and InCond-PI as green.

The performance indexes ITAE, IAE, ISE and ITSE are also computed for PV subsystem. These indexes are computed using e_PV_(t) = P_PV-ref_(t)-P_PV_(t) for HWNFC, ITSKC, TSKC and InCond-PI as shown in Fig 11. The HWNFC has lowest values of all the indexes.

**Fig 12:**
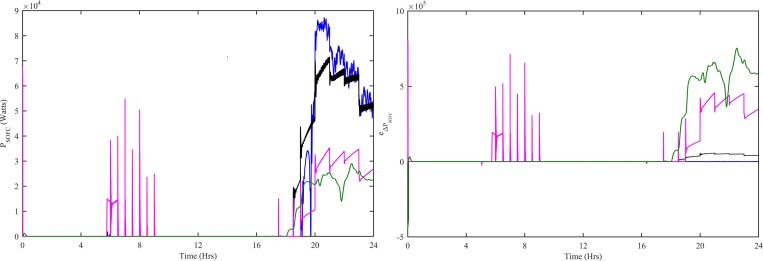
SOFC (a) Power (b) Power acquisition error with all four controllers. Ref. is designated as red, HWNFC as blue, ITSKC as black, TSKC Magenta and PI as green.

The power drawn from the SOFC depends upon the molar flow of input hydrogen. The molar flow of input hydrogen is controlled to get the swift response of SOFC. The HWNFC, ITSKC, TSKC and PI are used to get the swift response from SOFC. In the case of load variations, after a short transient period, the HWNFC quickly achieves the stable condition compared to the other controllers as shown in Fig 12 (A). The ITSKC, TSKC and PI take time and fluctuate more for load variations. The HWNFC has steady-state error = 410 Watts, % overshoot = 0 and % undershoot = -7. The ITSK has steady-state error = 16400 Watts, % overshoot = 21 and % undershoot = -12. The TSK has steady-state error = 49880 Watts, % overshoot = 368 and % undershoot = -4. The PI has steady-state error = 61890 Watts, % overshoot = 12.75 and % undershoot = 0. The HWNFC provides a better control than ITSKC, TSKC and PI as shown in Fig 12 (B).

**Fig 13:**
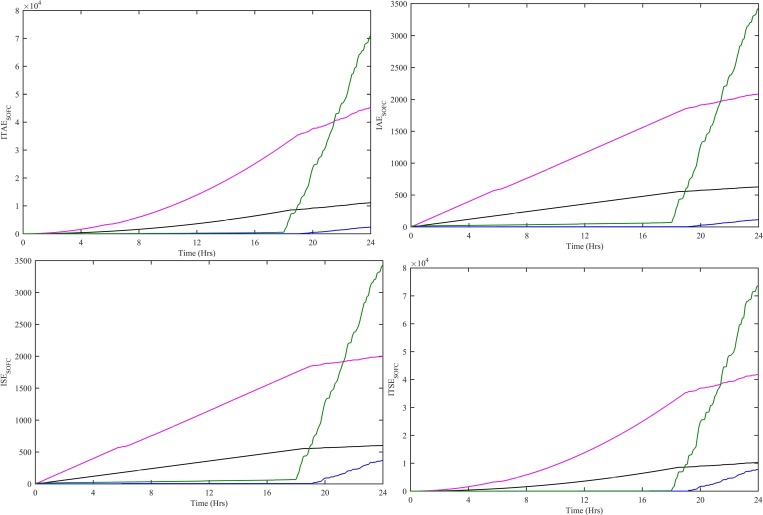
Performance indexes (a) ITAE, (b) IAE, (c) ISE and (d) ITSE for SOFC controllers. HWNFC is designated as blue, ITSKC as black, TSKC Magenta and PI as green.

The performance indexes ITAE, IAE, ISE and ITSE are computed for SOFC. These indexes are computed using e_SOFC_(t) = P_SOFC-ref_(t)-P_SOFC_(t) for HWNFC, ITSKC, TSKC and PI. The HWNFC indexes have least and flattest profile as shown in Fig 13. The HWNFC has a more stable output SOFC power as compared to other controllers.

**Fig 14:**
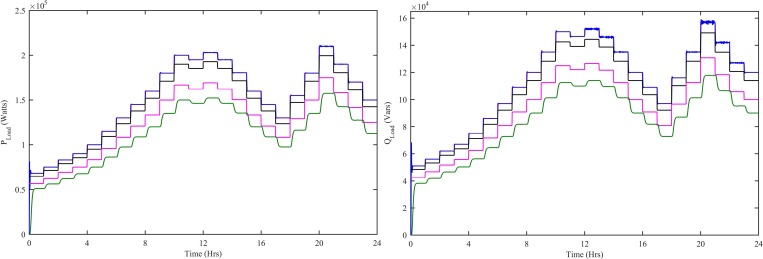
Load (a) Active power (b) Reactive Power with all four controllers. WNFC is designated as blue, ITSKC as black, TSKC Magenta and PI as green.

The reference power is shown in Fig 14 is the load required power. The load is actually the cumulative load of residential load and charging station load, i.e., P_Load_ = P_residenitial_+P_CS_. At each hour, the load required power is essentially satisfied by the power extracted from the generating sources used in the HPS. In Fig 14, the P_WNFC_ is the load power when the WT has CWNFC, PV has HWNFC and SOFC has HWNFC, whereas, the P_TSKC_ is the load power when the WT has ITSKC, PV has ITSKC and SOFC has ITSKC, the P_TSKC_ is the load power when the WT has TSKC, PV has TSKC and SOFC has TSKC and, the P_PI_ is the load power when the WT has Hyst-PI, PV has InCond-PI and SOFC has PI. The active power of the load provided by P_WNFC_ has 800 watts steady-state error, whereas, P_ITSKC_ has 10 kWatts, P_TSKC_ has 35 kWatts and P_PI_ has 50 kWatts. The reactive power of the load provided by Q_WNFC_ has 1000 watts steady-state error, whereas, Q_ITSKC_ has 7.2 kWatts, Q_TSKC_ has 25.3 kWatts and Q_PI_ has 36.5 kWatts. Moreover, the active and reactive powers of the load provided by P_WNFC_ and Q_WNFC_ have less fluctuations as compared to other controllers.

**Fig 15:**
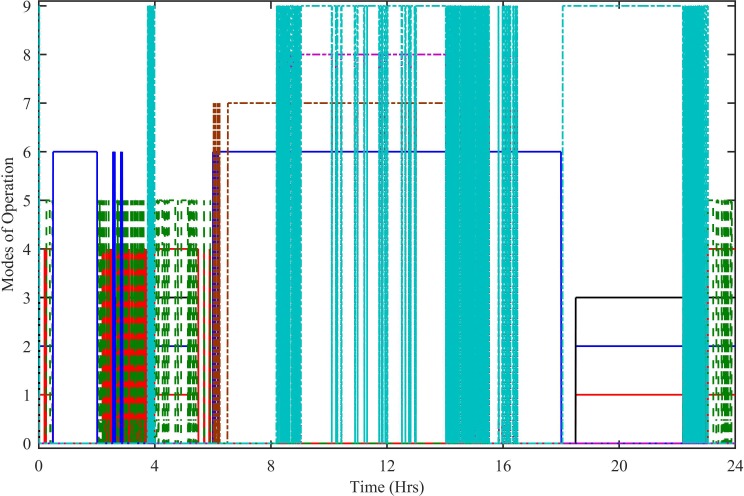
Modes of operation.

In the stated HPS, the continuous and reliable power supply to the load is ensured by using nine different modes of operation. Fig 15. Shows all the nine modes of operation. Mode 1 is activated during 0–0.4 hrs, 2–5.5 hrs and then again 18.5–24 hrs. During this mode of operation, the load power is met by the power taken from renewable sources and battery storage system. Mode 2 is activated during 0–0.5 hrs, 2–6 hrs and then again 18.5–24 hrs. In this mode, renewable sources, battery and SC deliver the power to the load. In mode 3, renewable sources, battery, SC and SOFC deliver the power to the load. In mode 4, the load power is met by the power taken from renewable sources, battery, SC, SOFC and utility grid. The mode 5 remains active during 0–0.5 hrs, 2.08–6 hrs and 22.25–24 hrs. During this mode of operation, the grid is unavailable due to having peak load hours, so, renewable sources, battery, SC, SOFC and MT deliver the power to the load. The mode 6 is activated during 0.5–2.015 hrs, 2.63 hrs, 2.84 hrs and 6–18 hrs. Mode 6, the HPS has excess power, because, the renewable sources generate more power than the load. This excess power is utilized by electrolyzer to produce hydrogen for SOFC. Mode 7 activates and deactivates during 6.02–16.49 hrs. During mode 7, again there is excess power which is used to charge the SC and then given to the electrolyzer. Mode 8 activates and deactivates during 8.75–16.49 hrs. In this mode of operation, the excess power is given to SC, utility grid and electrolyzer. Mode 9 is activated during 3.7–4 hrs, 8.2–15.52 hrs, 15.84–16.49 hrs and 18–23 hrs. During this mode of operation, the battery is disconnected and the excess power is utilized by SC, utility grid and electrolyzer.

**Fig 16:**
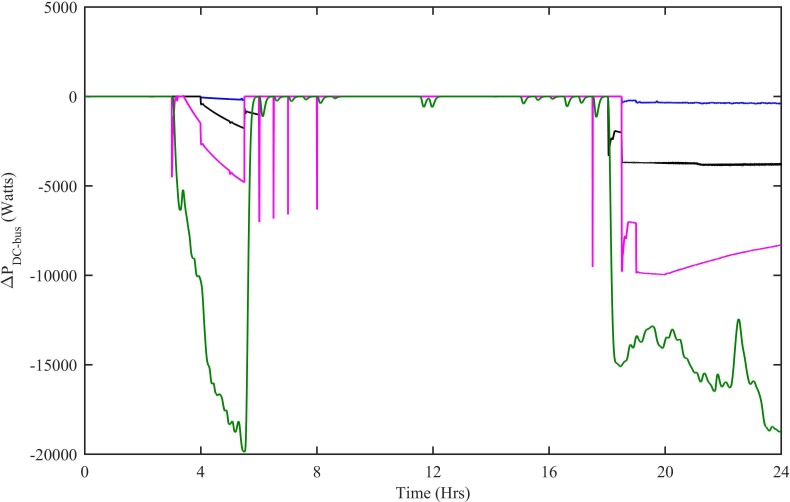
Net power on DC bus with all four controllers. WNFC is designated as blue, ITSKC as black, TSKC Magenta and PI as green.

In order to ensure the stability of HPS, the net power on DC as well as AC bus must be zero. Fig 16 shows the net power on the DC bus with all four controllers. The ΔP_WNFC_ is the net DC bus power when the WT has CWNFC, PV has HWNFC and SOFC has HWNFC, whereas, the ∆P_ITSKC_ is the net DC bus power when the WT has ITSKC, PV has ITSKC and SOFC has ITSKC, the ΔP_TSKC_ is the net DC bus power when the WT has TSKC, PV has TSKC and SOFC has TSKC and the ΔP_PI_ is the net DC bus power when the WT has Hyst-PI, PV has InCond-PI and SOFC has PI. The net power on DC bus is close to zero in ΔP_WNFC_ case.

**Fig 17:**
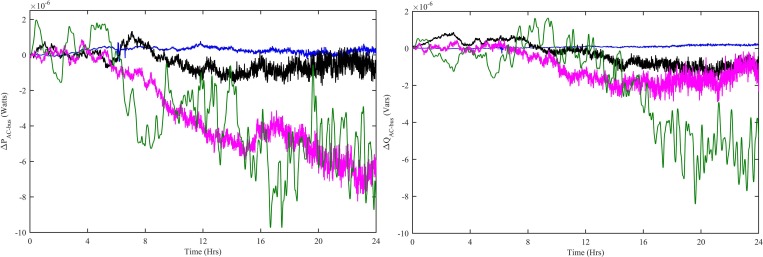
Net (a) Active power (b) Reactive power on AC bus with all four controllers. WNFC is designated as blue, ITSKC as black, TSKC Magenta and PI as green.

Similarly, the net active and reactive powers on AC bus are shown in Fig 17. Among all the four cases, the net active and reactive powers on AC bus are approximately zero in ΔP_WNFC_ and ΔQ_WNFC_ case.

**Fig 18:**
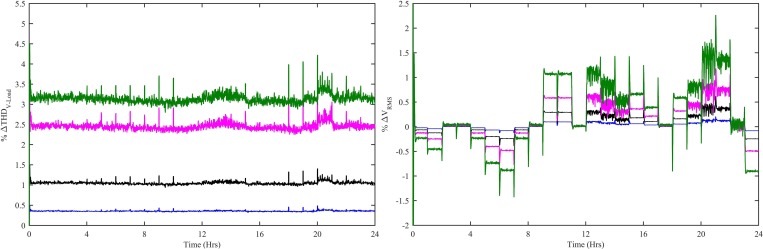
Percentage change in load (a) Voltage THDs (b) RMS voltage with all four controllers. WNFC is designated as blue, ITSKC as black, TSKC Magenta and PI as green.

Power quality is the most important aspect of the stated HPS. Power quality is particularly addressed in HPS, which is clearly shown in Fig 18. Total harmonic distortion (THD) for load voltage and load RMS voltage are all in their acceptable limits in all four cases, which ensures that the system is quite stable. The maximum deviations of voltage THD and RMS voltage are quite less in the case of THD_WNFC_ and V_RMS-WNFC_ as compared to other two cases.

The power conversion efficiency for WT, PV and SOFC is the ratio of its output power to the input power, over a specified operating period. The power conversion efficiency for WT, PV and SOFC can be either static or dynamic. The static efficiency refers to the stable conditions of wind speed, irradiance level and load, whereas, the dynamic efficiency is associated with the varying conditions of wind speed, irradiance levels and load. The dynamic efficiency of adaptive MPPT-VSWT-PMSG, MPPT-PV and SOFC tracking control system can be computed using the following equation:

$$\eta_{100}=\frac{\int_{t0}^{tf}Pdt}{\int_{t0}^{tf}P_{ref}dt}\times 100=\frac{\int_{t0}^{tf}\left(V\times I\right)dt}{\int_{t0}^{tf}P_{ref}dt}\times 100$$ (57)

**Fig 19:**
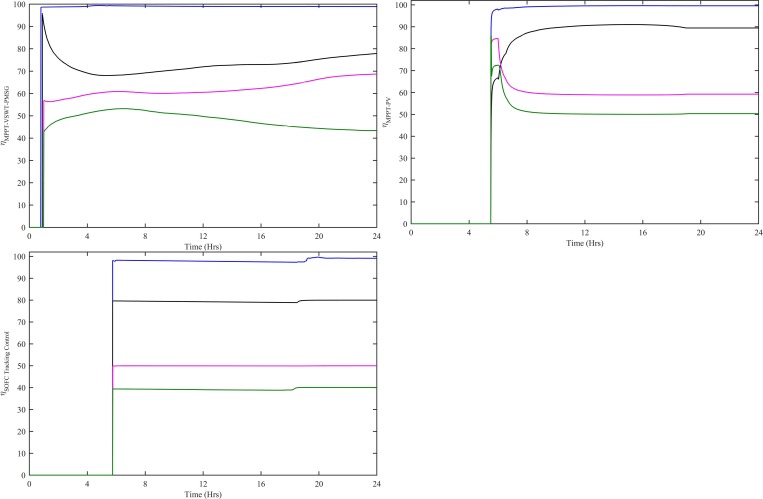
Dynamic efficiency for (a) MPPT-VSWT-PMSG (b) MPPT-PV (c) SOFC tracking control with all four controllers. WNFC is designated as blue, ITSKC as black, TSKC Magenta and PI as green.

Where η_100_ is the efficiency and P is P_WT-CWNFC_, P_PV-HWNFC_ and P_SOFC-HWNFC_ in the case of VSWT-PMSG, PV and SOFC, respectively. The dynamic efficiency for MPPT-VSWT-PMSG, MPPT-PV and SOFC tracking control with all four controllers is shown in Fig 19. The dynamic efficiency for MPPT-VSWT-PMSG with CWNFC has 98.82%, ITSKC has 77.76%, TSKC has 68.65% and Hyst-PI has 43.4% at t = 24 hrs. Similarly, the dynamic efficiency for MPPT-PV with HWNFC has 99.6%, ITSKC has 89.44%, TSKC has 59.24% and InCond-PI has 50.35% at t = 24 hrs. The dynamic efficiency for SOFC tracking control with HWNFC has 99.14%, ITSKC has 80%, TSKC has 49.99% and PI has 40.05% at t = 24 hrs.

**Fig 20:**
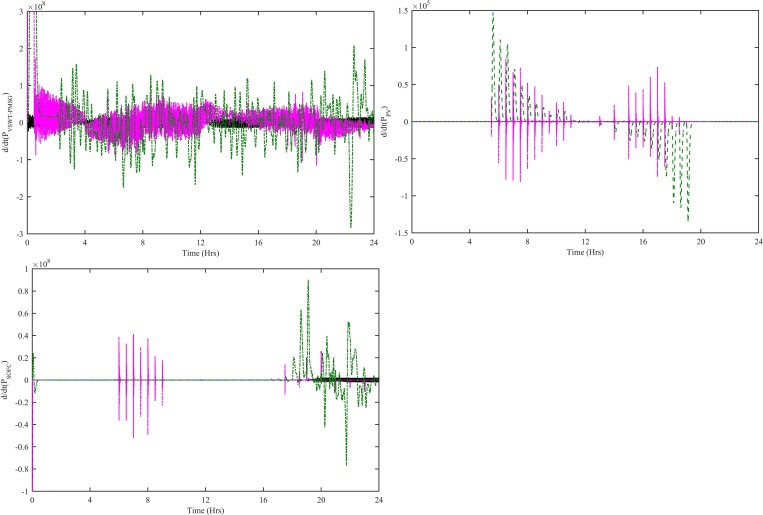
Rate of change of (a) VSWT-PMSG power (b) PV power (c) SOFC power with all four controllers. WNFC is designated as blue, ITSKC as black, TSKC Magenta and PI as green.

The dynamic efficiency based on the proposed MPPT-VSWT-PMSG, MPPT-PV and SOFC tracking control is maximum throughout the simulation time. This implies that the proposed adaptive control paradigms extract maximum power as compared to the other control schemes. Fig 20 shows that the rate of change for (a) VSWT-PMSG power (b) PV power (c) SOFC power computed with CWNFC, ITSKC and TSKC. The rate of change for VSWT-PMSG power, PV power and SOFC power can be computed as follows:

$$\frac{dP}{dt}={\frac{P(t)-P(t-1)}{\Delta t}|}_{t>(t-1)}$$ (58)

Where P is P_WT-CWNFC_, P_PV-HWNFC_ and P_SOFC-HWNFC_ in the case of VSWT-PMSG, PV and SOFC, respectively. The rate of change is minimum in VSWT-PMSG power using CWNFC, PV power using HWNFC and SOFC power using HWNFC. The rate of change of the powers extracted by the proposed control paradigms remains minimum during simulation. It clearly indicates that the converters switches will be subjected to minimum stress in case of the proposed adaptive control schemes.

## 6 Conclusion

This paper presents the integration of VSWT-PMSG, PV, SOFC, electrolyzer, MT along with storage systems (battery and SC) in a grid-connected HPS to meet the cumulative load of residential load and charging station load. The stated HPS is a large-scaled nonlinear model of various subsystems. In order to improve the performance of the HPS, three different wavelets based indirect adaptive NeuroFuzzy control schemes are used which is the main contribution of this research work. A Chebyshev wavelet embedded NeuroFuzzy indirect adaptive MPPT control scheme is used to extract the optimum power from VSWT-PMSG. A Hermite wavelet embedded NeuroFuzzy indirect adaptive control scheme is used to acquire the optimum power from PV system. Moreover, the load following issue of SOFC is solved using a Hermite wavelet incorporated NeuroFuzzy indirect adaptive control scheme. To maintain a balance between the generation and load in the HPS, a supervisory control policy which consists of nine different modes of operation is used. To validate the performance, the proposed control techniques are tested in a Matlab/Simulink environment. The proposed control paradigms have higher precision than ITSKC, TSKC and PI in terms of efficiency, steady-state error and performance indexes. The effectiveness of the proposed schemes is confirmed by the simulation results.

## Future work

The future work is to replace the conventional PID controllers by adaptive PID controllers used to control the converters more effectively.
