# Correction: Indirect adaptive soft computing based wavelet-embedded control paradigms for WT/PV/SOFC in a grid/charging station connected hybrid power system

**DOI:** 10.1371/journal.pone.0195914

**Published:** 2018-04-11

**Authors:** Sidra Mumtaz, Laiq Khan, Saghir Ahmed, Rabiah Badar

The fourth author’s name is spelled incorrectly. The correct name is: Rabiah Badar. The correct citation is: Mumtaz S, Khan L, Ahmed S, Badar R (2017) Indirect adaptive soft computing based wavelet-embedded control paradigms for WT/PV/SOFC in a grid/charging station connected hybrid power system. PLoS ONE 12(9): e0183750. https://doi.org/10.1371/journal.pone.0183750.

The affiliation for the fourth author is incorrect. Rabiah Badar is not affiliated with #1 but with #2 Department of Electrical Engineering, COMSATS Institute of Information Technology, Islamabad, Pakistan.
